# Correlation of preoperative CT imaging shift parameters of the lateral plateau with lateral meniscal injury in Schatzker IV-C tibial plateau fractures

**DOI:** 10.1186/s12891-023-06924-7

**Published:** 2023-10-06

**Authors:** Yulong Liu, Run Fang, Bizhi Tu, Zheng Zhu, Chengnan Zhang, Rende Ning

**Affiliations:** Department of Orthopedics, The Third Affiliated Hospital of Anhui Medical University, The First People’s Hospital of Hefei, 390 Huaihe Road, Hefei, 230061 Anhui China

**Keywords:** Schatzker IV-C tibial plateau fracture, Lateral meniscus injury, Depression, Widening

## Abstract

**Background:**

Schatzker IV-C is a high-energy tibial plateau fracture often accompanied by lateral meniscus injuries. While imaging examinations are routine preoperative measurements, the correlation between CT imaging shift parameters of the lateral plateau and lateral meniscal injury in Schatzker IV-C fractures remains uncovered.

**Methods:**

This retrospective study enrolled a total of 60 patients with Schatzker IV-C tibial plateau fractures at the First People’s Hospital of Hefei. Prior to surgery, CT imaging was used to measure the numerical values of lateral plateau depression (LPD) and lateral plateau widening (LPW). The degree of lateral meniscus injury was confirmed based on intraoperative direct vision, with patients being classified into meniscus injury and non-meniscus injury groups. Dichotomous logistic regression was employed to evaluate the correlation between LPD, LPW, and lateral meniscus injury, while the optimal cut-off points for predicting lateral meniscal injury with LPD and LPW were determined using receiver operator characteristic (ROC) curves.

**Results:**

The meniscus injury group exhibited a mean LPD of 15.3 ± 3.5 mm, which was significantly higher than the non-meniscus injury group’s mean LPD of 8.4 ± 3.4 mm (P < 0.05). Similarly, the meniscus injury group had a larger mean LPW of 9.4 ± 1.8 mm compared to the non-meniscus injury group’s mean LPW of 6.9 ± 0.9 mm (P < 0.05). The optimal cut-off points for predicting lateral meniscal injury were determined to be 8.40 mm for LPD (with a sensitivity of 95%, specificity of 85%, and AUC of 0.898) and 7.90 mm for LPW (with a sensitivity of 75%, specificity of 90%, and AUC of 0.897).

**Conclusions:**

Patients with Schatzker IV-C tibial plateau fractures are at a significantly higher risk of lateral meniscal injury when the LPD exceeds 8.40 mm and/or the LPW exceeds 7.90 mm. Our results may provide novel reference metrics for the early diagnosis of lateral meniscal injury in Schatzker IV-C tibial plateau fracture patients when the MRI examination is not available.

**Supplementary Information:**

The online version contains supplementary material available at 10.1186/s12891-023-06924-7.

## Introduction

The classification of tibial plateau fractures encompasses various categories, with the Schatzker Staging System being widely accepted as the most commonly used clinical method [1–3]. Schatzker IV is a relatively uncommon form of medial tibial plateau fracture characterized by splitting or depression of the articular surface. This type of fracture represents approximately 10–30% of all tibial plateau fractures among patients [4, 5]. Based on the relationship between the fracture line and the intercondylar eminence, Wahlquist et al. [6] further differentiated Schatzker IV fractures into three subcategories, namely subtypes A, B, and C fractures. Type A fractures have the fracture line located on the medial side of the eminence, type B fractures have the fracture line on the intercondylar eminence, and type C fractures have the fracture line on the lateral side of the eminence. Schatzker IV-C fracture is characterized by severe injury with significant force, often accompanied by dislocation, lateral meniscus entrapment, and various ligament injuries [5, 7]. In their study, Barrow et al. found that meniscus injury incidence in Schatzker IV tibial plateau fractures was 25% [8]. Furthermore, other studies have reported occurrence rates of 63% for lateral meniscus tears and 44.4% for medial meniscus tears [9]. Because the location of the fracture line influences the severity of soft tissue injury, with a higher prevalence of lateral meniscus entrapment when the fracture line is closer to the lateral side [6, 9], Schatzker IV-C fractures-related lateral meniscus injury will significantly increase the contact stress and instability in the knee joint, which results in the development of traumatic arthritis and joint stiffness [10–13]. Therefore, timely diagnosing and treating the meniscus injury after Schatzker IV-C fractures is imperative to achieve optimal patient outcomes.

Although magnetic resonance imaging (MRI) has a high diagnostic accuracy for assessing meniscal and ligament injuries, and is often considered the gold standard for preoperative soft tissue diagnosis [14], MRI examinations have lengthy wait times and high costs. The scanning process is time-consuming, and there is limited availability of MRI [15, 16]. Moreover, acute tibial plateau fractures can cause diffuse soft tissue edema around the knee joint, potentially resulting in the overdiagnosis of meniscal injuries [17]. Additionally, preoperative scanning is not commonly performed, and it lacks widespread adoption in primary healthcare facilities in our country [17–19]. Meanwhile, CT imaging can assist orthopedic surgeons in accurately assessing lateral plateau depression through three-dimensional reconstruction techniques. Therefore, the preferred preoperative examination for acute tibial plateau fractures is CT rather than MRI [20]. CT scans have been widely adopted for assessing various tibial plateau fractures and obtained detailed fracture-related information, including the fracture line location, the extent of fracture block fragmentation, and any changes in the displacement of the lateral plateau’s articular surface [21, 22]. In recent years, growing evidence shows that preoperative knee X-rays and CT imaging parameters can effectively predict the presence of combined soft tissue injuries, particularly lateral meniscal injuries, in patients with tibial plateau fractures [17, 23–27]. However, the correlation between preoperative CT imaging shift parameters of the lateral plateau and lateral meniscal injury in Schatzker IV-C tibial plateau fractures remains unknown.

In this study, we retrospectively analyzed preoperative CT scans and surgical observations in patients with Schatzker IV-C fractures. Our findings present a methodology for predicting lateral meniscal injuries in Schatzker IV-C fractures, which may aid primary-level hospitals without access to MRI or patients unable to undergo MRI testing in promptly diagnosing and treating meniscus injuries following Schatzker IV-C fractures.

## Materials and methods

### General data

From February 2012 to August 2022, we retrospectively reviewed the medical records and imaging data of 70 patients with Schatzker IV tibial plateau fractures at the Trauma Orthopedic Center of the First People’s Hospital in Hefei. Our study was approved by the hospital ethics committees. Inclusion Criteria: (1) Patients 18 years of age or older with a clear history of knee trauma and a confirmed diagnosis of Schatzker IV-C tibial plateau fracture on X-ray and CT scans, based on the Schatzker and Wahlquist classification system. (2) Individuals who underwent surgery within 2 weeks of their injury. (3) Patients without a history of knee pain, surgery, or dysfunction, and without any prior history of gout or arthritis. Exclusion Criteria: (1) Patients with severe bone metabolic diseases, pathological fractures, and similar conditions. (2) Individuals with fractures around the same-side limb knee joint. (3) Patients who did not undergo preoperative CT examination or open reduction internal fixation (ORIF).

A total of 60 patients with Schatzker IV-C tibial plateau fractures met the inclusion criteria. All fracture patients undergo a postoperative followed at least one year, during which the Hospital for Special Surgery (HSS) Knee Score was recorded. A higher score indicates better knee function. The clinical efficacy is classified as follows: excellent (> 85 points), good (70–84 points), moderate (60–69 points), and poor (< 59 points). The same experienced orthopedic surgeon recorded the patient’s general clinical information, medical history, and surgical outcomes, including their gender, age, mechanism of injury, and past medical history.

### Surgery

#### Surgical objectives

All patients underwent preoperative X-rays and CT scans to determine the extent of the injury of Schatzker IV-C fracture. The patients were treated with ORIF through a posterior medial approach and an additional lateral parapatellar approach, using support plates and screws. The goal was to reconstruct the tibial joint plane, reduce step formation, restore the mechanical axis, achieve stable fracture repositioning and internal fixation, and repair the damaged soft tissues around the knee as much as possible to minimize the risk of post-traumatic osteoarthritis.

#### Surgical Procedure steps

**Fig. 1:**
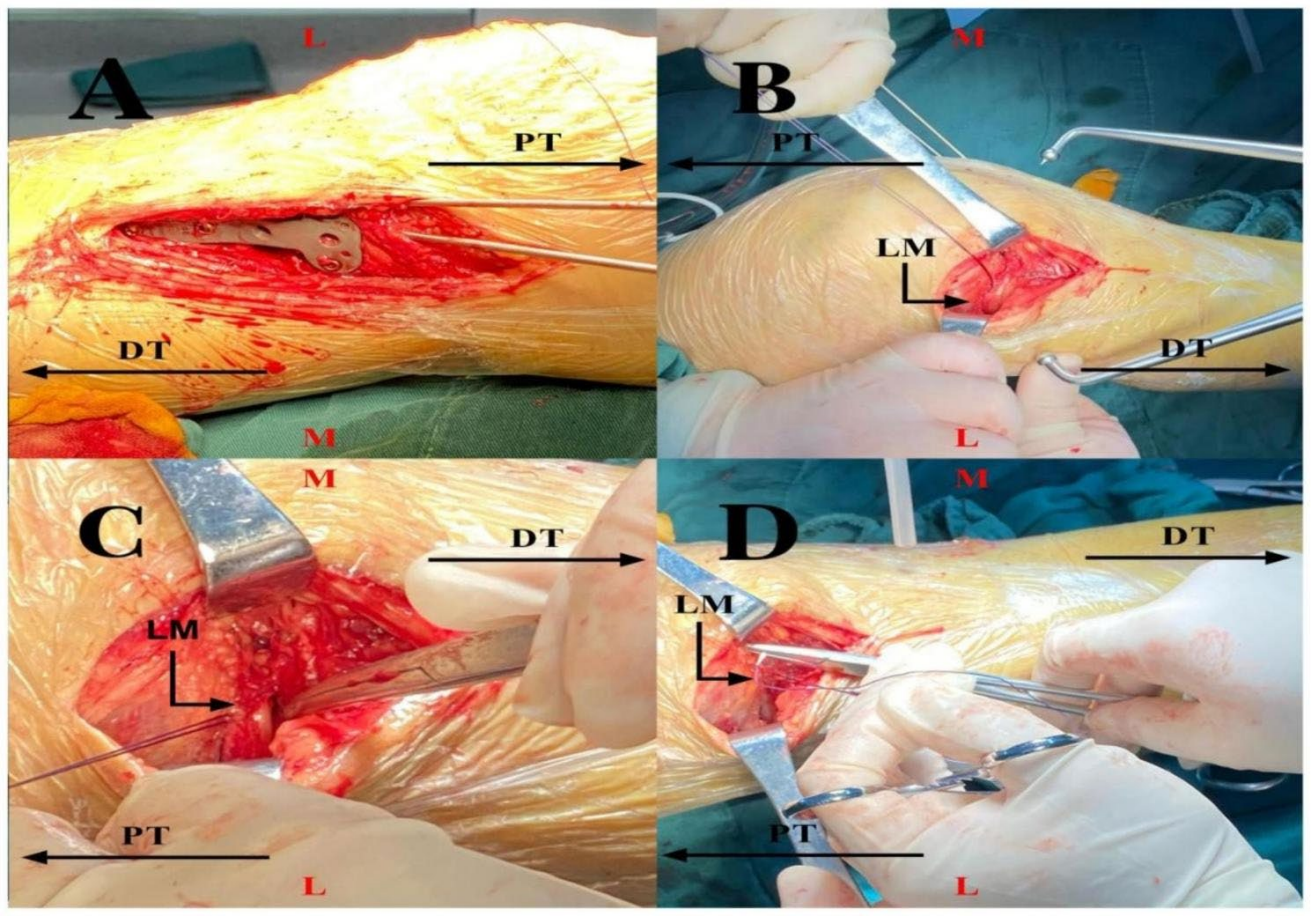
The surgical steps of using a posterior medial approach and an additional lateral parapatellar approach to address the fracture and the entrapment of the lateral meniscus. (A) Reduction and fixation of the fracture fragments using a locking plate through a posterior medial approach. (B) Creation of an additional incision near the lateral patellar border to adequately expose the lateral meniscus. (C) Repositioning the displaced lateral meniscus to its original anatomical position. (D) Suturing the lateral meniscus to the joint capsule. L, Lateral; M, Medial; PT, Proximal Tibia; DT, Distal Tibia; LM, Lateral Meniscus

The patient is placed in a supine position, and a pneumatic tourniquet is applied to the proximal thigh. Firstly, the joint capsule is incised using a posterior medial approach to expose the collapsed medial tibial plateau fracture fragment adequately. Attempt to reduce the fracture, if reduction is difficult, it may indicate entrapment of the lateral meniscus within the fracture line. In such cases, exploratory reduction of the entrapped lateral meniscus can be performed through the posterior medial approach. If reduction fails, an additional lateral parapatellar approach is taken to expose the lateral tibial plateau articular surface. Under direct visualization, a meniscal hook probe is used to assess the specific condition of the lateral meniscus injury, including examining the tibial and femoral surfaces of the meniscus. If necessary, the condition of the medial meniscus and other soft tissue injuries, such as ligaments, should also be evaluated. If entrapment of the lateral meniscus is identified, it can be grasped with Allis forceps and pulled laterally for reduction under direct visualization, aiming to relocate the meniscus from within the joint back to its original anatomical position. Next, the medial tibial plateau fracture fragment is anatomically reduced using a posterior medial approach. Simultaneously, the collapsed key fracture fragment is elevated and artificial bone material is implanted at the collapsed site. The fragment is temporarily stabilized using Kirschner wires to restore the flatness of the joint surface. If the key fracture fragments are the tibial-side attachment points of the anterior cruciate ligament or the posterior cruciate ligament, it is reinforced with a hollow screw for fixation. Once satisfactory reduction is achieved, apply internal fixation using a medial anatomical plate for compression fixation. Finally, use a size 0 absorbable suture to reattach the torn lateral meniscus to the joint capsule. Figure 1 shows the surgical steps of using a posterior medial approach and an additional lateral parapatellar approach to address the fracture and the entrapment of the lateral meniscus.

### Observation index

**Fig. 2:**
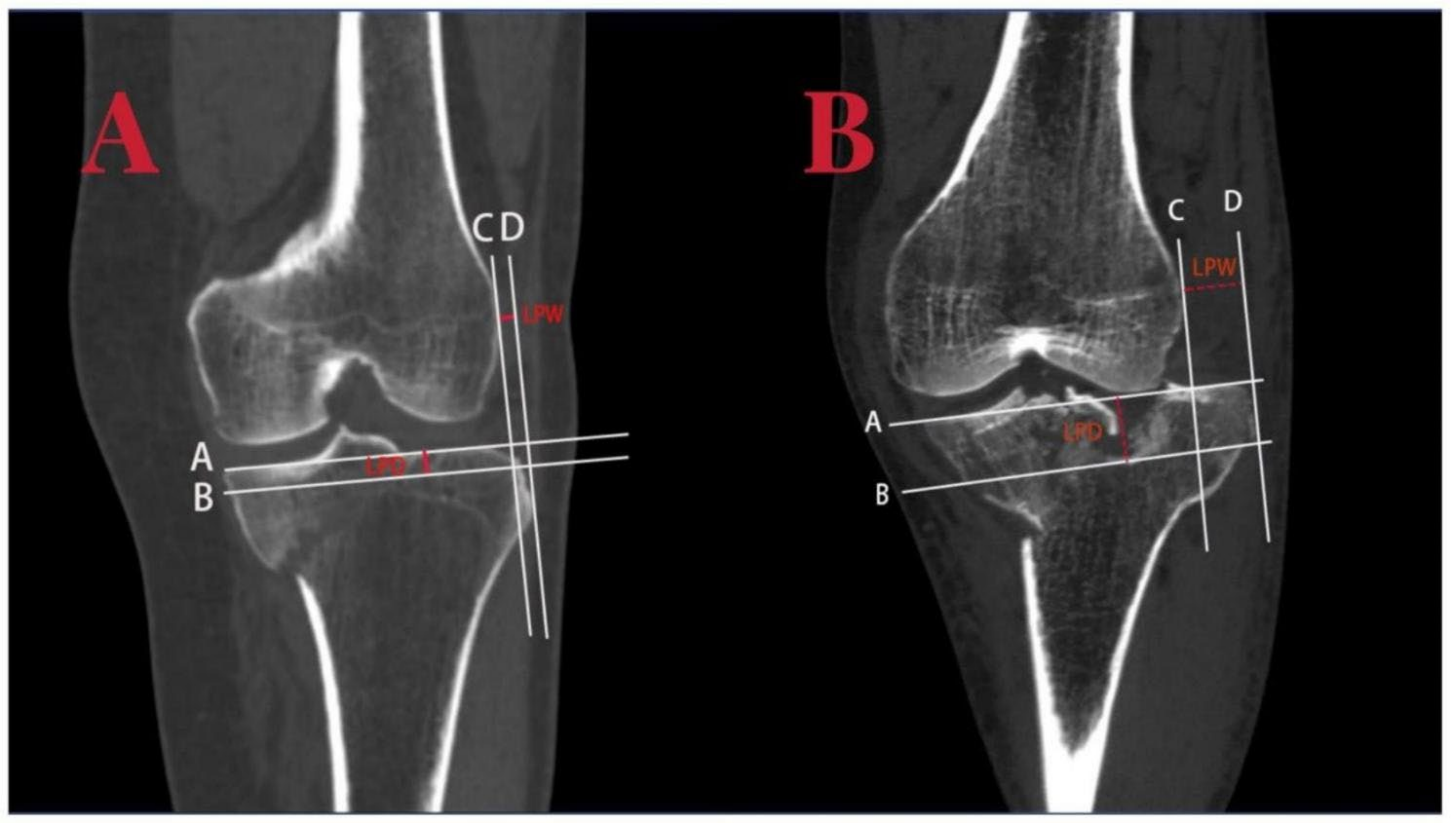
Measurement of lateral plateau depression (LPD) and lateral plateau widening (LPW) in the CT Coronal Scan. Example A: A 52-year-old male patient with a Schatzker IV-C Tibial Plateau Fracture and no lateral meniscus injury; the LPD measures 7.84 mm and the LPW measures 4.65 mm. Example B: A 50-year-old female patient with a Schatzker IV-C Tibial Plateau Fracture and lateral meniscus injury; the LPD measures 20.50 mm and the LPW measures 12.68 mm

All preoperative CT scans of Schatzker IV-C fractures were reviewed and quantified by two seasoned orthopedic surgeons blinded to the patient’s clinical examination reports and surgical findings. The following metrics were evaluated: 1.the amount of lateral plateau depression (LPD) and lateral plateau widening (LPW); 2.the average LPD and LPW, minimum LPD, and minimum LPW (all in millimeters); 3.the presence of lateral meniscus injury, which was determined through intraoperative inspection. The patients were categorized into two groups based on lateral meniscus injury. The two surgeons each performed the measurements three times at a given time and calculated an average for the final analysis. The methodology for measurement is illustrated in Fig. 2.

LPD: A horizontal line is drawn parallel to the articular surface from the lowest point of the medial plateau, called Line (A) Another horizontal line is drawn parallel to Line A from the lowest point of the lateral plateau depression, called Line (B) The vertical distance between Line A and Line B is measured and defined as LPD.

LPW: A vertical line is drawn from the outermost point of the lateral femoral condyle, called Line C. Another line is drawn parallel to Line C from the outermost point of the lateral tibial plateau, called Line D. The horizontal distance between Line C and Line D is measured and defined as LPW.

### Statistical analysis

In this study, SPSS 26.0 statistical software was utilized to analyze all data. Categorical data regarding patient demographics were assessed using the chi-square test. Numerical variables, such as age, LPD, and LPW, were assessed for normal distribution and presented as mean ± standard deviation using t-tests. The Mann-Whitney-U test was used to determine statistical significance for non-normally distributed data. Logistic regression was employed to investigate the effect of LPD and LPW on the probability of lateral meniscus injury. Receiver operator characteristic (ROC) curves were constructed to evaluate the minimum LPD and LPW that could lead to a lateral meniscus injury. Area under the curve (AUC) analysis was used to calculate the reliability of LPD and LPW in early predicting the lateral meniscus injury. A statistical difference was determined as P < 0.05.

Intra- and interobserver reliability was evaluated using intraclass correlation coefficients (ICC) with confidence intervals (CI), Based on the 95% confident interval of the ICC estimate, values less than 0.5, between 0.5 and 0.75, between 0.75 and 0.9, and greater than 0.90 are indicative of poor, moderate, good, and excellent reliability, respectively.

## Results

### Patients’ information

A total of 60 patients with Schatzker IV-C tibial plateau fractures were included in this study. Detailed information regarding local soft tissue injuries of all fracture patients is presented in Table S1, and the results of their one-year postoperative Hospital for Special Surgery (HSS) Knee Score are presented in Table S2. Among the included patients, 24 cases of fractures resulted from motor vehicle accidents, 15 from pedestrian-motor vehicle accidents, ten from pedestrian-electric vehicle accidents, 9 from falls at heights, and two from falls on the ground. During the surgery, a concomitant lateral meniscus injury was observed in 40 patients (66.7%), with 27 cases (45%) exhibiting lateral meniscus entrapment. Additionally, 20 patients (33.3%) did not present any lateral meniscus damage. Herein, 40 patients with the lateral meniscus injury were included as the experimental group, and 20 patients were included in the non-lateral meniscus injury group as the control group. There were no statistically significant differences between the two groups in terms of gender, age, or left-right side (P > 0.05). The detailed information is presented in Table 1.

**Table 1:** Comparison of general information and coronal CT findings between meniscal injury group and non-injury group

	Meniscus injury group	Non-meniscus injury group	*P* value
Patients, No. (%)	40(66.7%)	20(33.3%)	
Age, y (mean ± SD)	45.8 ± 18.2	44.5 ± 16.5	*P* > 0.05
Gender, No. (Males: Females)	22: 18	13: 7	*P* > 0.05
Side, No. (Left: Right)	24: 16	8: 12	*P* > 0.05
LPD, mm (mean ± SD)	15.3 ± 3.5	8.4 ± 3.4	*P* < 0.001
LPW, mm (mean ± SD)	9.4 ± 1.8	6.9 ± 0.9	*P* < 0.001

Abbreviations: No. Number; y years; SD Standard deviation; LPD Lateral plateau depression; LPW Lateral plateau widening

### The difference in lateral plateau shift between the experimental and control group

Excellent intra-observer reliability (Observer 1, ICC = 0.910, p < 0.001, 95% CI 0.891–0.924; Observer 2, ICC = 0.915, p < 0.001, 95% CI 0.894–0.929) was found in LPD, and good intra-observer reliability (Observer 1, ICC = 0.881, p < 0.001, 95% CI 0.865–0.904; Observer 2, ICC = 0.892, p < 0.001, 95% CI 0.870–0.912) was found in LPW. Both the LPD (ICC = 0.895, p < 0.001, 95% CI 0.871–0.911) and LPW (ICC = 0.876, p < 0.001, 95% CI 0.855–0.901) had good inter-observer reliabilities. For the meniscus injury group, the minimum LPD was 6.88 mm, the maximum was 20.85 mm, and the mean LPD was 15.3 ± 3.5 mm. The non-meniscus injury group had a minimum LPD of 5.68 mm, maximum LPD of 16.96 mm, and mean LPD of 8.4 ± 3.4 mm. The difference in mean LPD between the two groups was statistically significant (P < 0.001). The mean LPW of the meniscus injury group was 9.4 ± 1.8 mm (range 6.80 to 13.37 mm), and the mean LPW of the non-meniscus injury group was 6.9 ± 0.9 mm (range 5.37 to 8.77 mm), with a statistically significant difference (P < 0.001).

To investigate the influencing factors of lateral meniscus injury, we employed binary logistic regression to assess the impact of depression and widening on lateral meniscus injury. The results revealed that both depression and widening showed statistical significance (P < 0.05), indicating that they are potential risk factors for lateral meniscus injury (Table 2).

**Table 2:** Logistic regression evaluated the effect of collapse and widening on lateral meniscus injury

	B	S.E.	Wald	*P*	OR	95%CI for OR	
						Lower	Upper
LPD	0.420	0.144	8.541	0.003	1.521	1.148	2.016
LPW	1.833	0.698	6.896	0.009	6.252	1.592	24.553

Abbreviations: LPD Lateral plateau depression; LPW Lateral plateau widening

### The lateral plateau shift parameter of CT can predict lateral meniscus injury

**Fig. 3:**
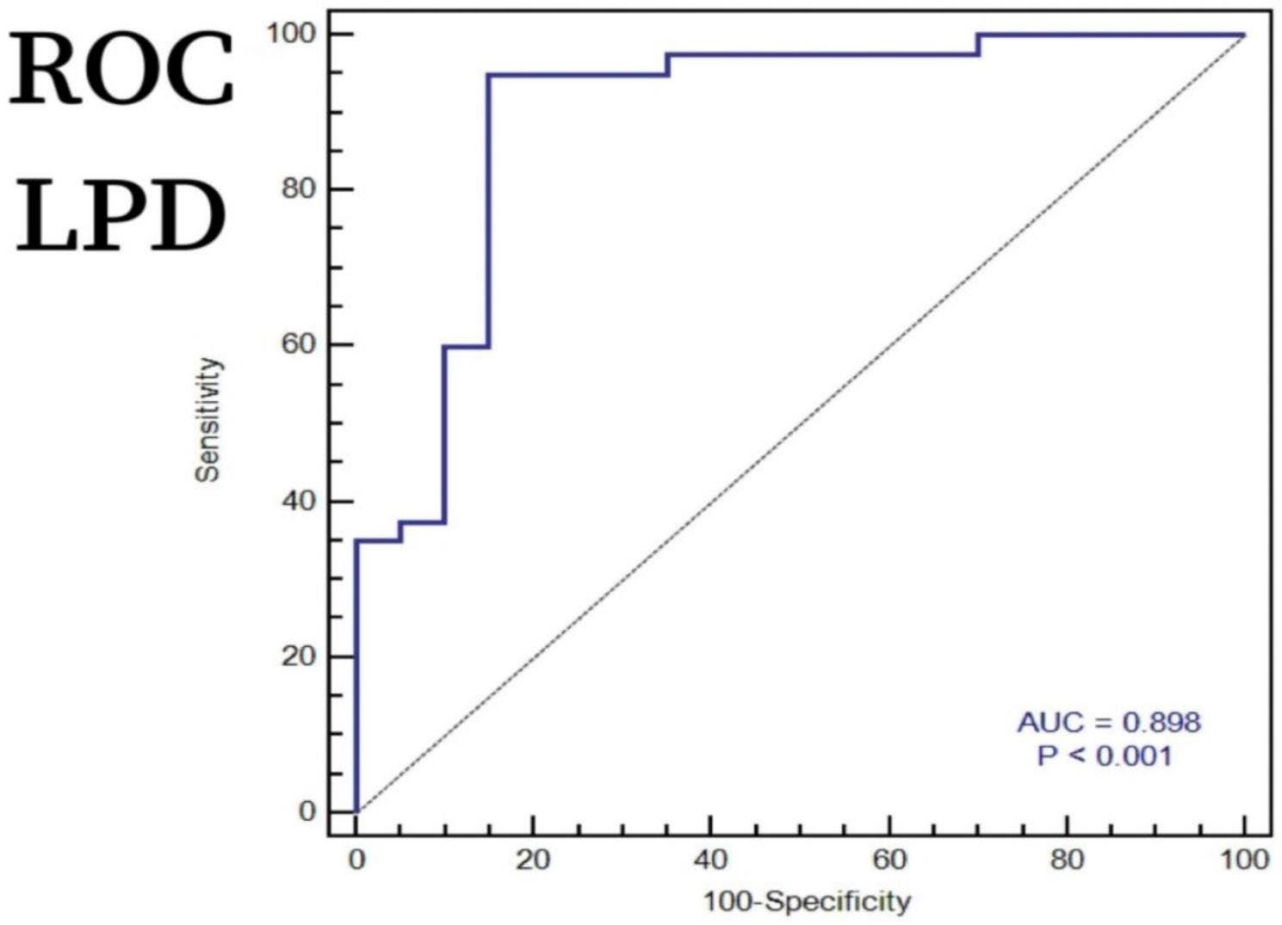
An ROC analysis was conducted using LPD to predict lateral meniscus injury in Schatzker IV-C tibial plateau fractures

**Fig. 4:**
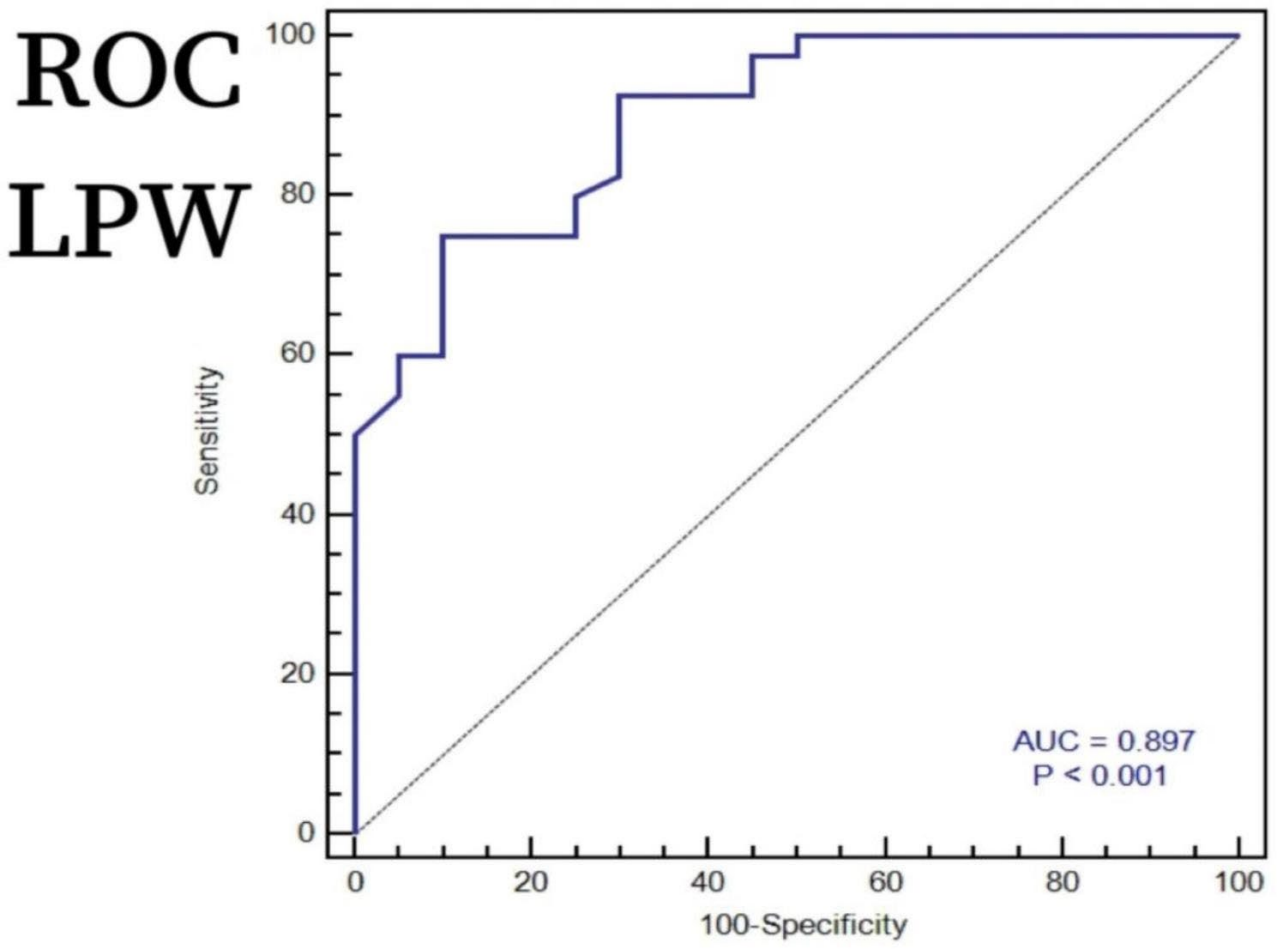
An ROC analysis was conducted using LPW to predict lateral meniscus injury in Schatzker IV-C tibial plateau fractures

To investigate whether preoperative CT imaging shift parameters of the lateral plateau can predict the occurrence of lateral meniscal injury in Schatzker IV-C tibial plateau fractures, we conducted ROC analysis to determine the diagnostic ability of optimal preoperative CT imaging shift parameters for lateral meniscus injury. Based on our findings, LPD was determined to have a predictive threshold of 8.40 mm (with a sensitivity of 95%, specificity of 85%, and AUC of 0.898) as shown in Fig. 3, while LPW had a predictive threshold of 7.90 mm (with a sensitivity of 75%, specificity of 90%, and AUC of 0.897) as illustrated in Fig. 4.

## Discussion

The primary objective of this retrospective study was to explore the correlation between preoperative CT imaging shift parameters of the lateral plateau and lateral meniscal injury in Schatzker IV-C tibial plateau fractures. Our findings revealed that lateral platform displacement in Schatzker IV-C fractures is closely linked to a higher risk of lateral meniscal injury. Preoperative CT measurements of LPD and LPW can be particularly useful in predicting Schatzker IV-C fracture-related meniscal injury and providing valuable clinical guidance.

Previous studies have primarily focused on Schatzker classification types other than Schatzker IV-C [19, 26–28]. Soft tissue injuries in Schatzker IV fractures are typically identified through physical examination, MRI, and arthroscopic exploration [14]. However, the application of these examinations during the acute injury phase remains limited. MRI is commonly used for detecting soft tissue injuries, but it excludes patients with contraindications such as cardiac pacemakers, defibrillators, metal implants, severe anxiety, or claustrophobia [29]. Arthroscopy, considered the gold standard for soft tissue damage detection following knee trauma, is expensive, technically complex, and associated with risks and complications such as extravasation of irrigation fluid, compartment syndrome, infection, nerve and vascular injuries [14, 30–33]. Herein, the application of arthroscopy may not be feasible for primary-level hospitals in developing countries. Moreover, arthroscopic surgery is explicitly contraindicated for complex tibial plateau fractures, such as IV-C fractures [34]. To address these examinations’ limitations, we adopted intraoperative direct visualization for precise and definitive information on meniscus injuries. Additionally, considering the impact of anatomical tibial slope on X-ray examination, we utilized coronal plane CT scans to obtain more objective measurements [35]. These distinctions set our study apart from previous research.

The incidence of tibial plateau fracture-related meniscal injury ranges from 21 to 99%, with lateral meniscal injury being the predominant type. Bennett et al. [36] reported three meniscal damage cases among nine patients (33.3%) with type IV tibial plateau fractures. Stannard et al. [37] noted a strong association between high-energy tibial plateau fractures and soft tissue injuries. They observed one medial meniscal tear and 5 cases of lateral meniscal tear among 13 patients with Schatzker type IV fractures. Michael et al. [38] observed that medial meniscal tears were commonly present in Schatzker type IV tibial plateau fractures (86%). Bingshan Yan et al. [9] found that lateral meniscal tears in Schatzker type IV tibial plateau fractures were 63%, while medial meniscal tears were 44.4%. Although meniscal injury in Schatzker type IV fractures has been previously documented, few studies have reported the incidence of tissue injuries following Schatzker type IV-C fractures. In the current study, we reported the possibility of ACL injury (86.7%), lateral meniscal injury (66.7%), medial meniscal injury (41.7%), partial posterior cruciate ligament (PCL) injury (63.3%), the incidence of lateral collateral ligament (LCL) injury (65%), and medial collateral ligament (MCL) injury (31.7%). Our results may complement the relationship between Schatzker type IV-C fracture and soft tissue injury.

This study highlights the close association between lateral plateau displacement and lateral meniscus injury in Schatzker IV-C fractures. Although, Mustonen et al. [18] introduce that there is no correlation between meniscal damage and the type or degree of depression in tibial plateau fractures, increasing reports suggest a higher incidence of soft tissue injuries in patients with a more significant displacement of the tibial lateral plateau fracture [19, 23, 25, 26]. To identify reliable indicators for early prediction of associated soft tissue injuries, researchers have focused on the correlation between preoperative X-ray and CT measurements and lateral meniscus damage [25, 39]. Gardner et al. [40] found 83% of patients exhibiting lateral meniscus injury on MRI once LPD length more than 6 mm and width exceeding 5 mm on preoperative X-ray measurements among 62 patients with Schatzker II fractures. Durakbasa et al. [39] found that LPD ≥ 14 mm and/or LPW ≥ 10 mm was correlated to the high risk of lateral meniscus tear based on preoperative X-rays measurements among 20 patients with Schatzker II tibial plateau fractures. Ringus et al. [41] measured LPD using coronal CT scans in 85 patients with Schatzker I-VI tibial plateau fractures, revealing an 8-fold increased risk for lateral meniscus tear with LPD ≥ 10 mm. Tang et al. [25] demonstrated that LPD exceeding 11 mm on preoperative CT scans was accompanied by 70.3% lateral meniscus injury among 132 patients with acute tibial plateau fractures (including 8 Schatzker IV fractures). However, they did not reveal the correlation between LPW measurements and meniscal tears. Kolb et al. [17] compared preoperative LPW measurements obtained from CT scans with MRI results and found 40% increased lateral meniscus tear risk with every LPW exceeding 1 mm among 55 patients with Schatzker I-III tibial plateau fracture. Hengrui Chang et al. [28] investigated CT imaging parameters and arthroscopic findings in 102 patients with acute tibial plateau fractures (including 22 cases of Schatzker IV fractures). They found a positive correlation between LPD > 6.3 mm and the risk of lateral meniscus tear. Salari et al. [26] utilized CT measurements and intraoperative visualization and found a 21% increased risk of meniscal tear with each 1 mm increase in maximum articular surface depression/displacement (AID) in 70 patients with Schatzker I-II tibial plateau fractures. Meanwhile, they also found 100% lateral meniscus tear when AID exceeds 4.3 mm. Ying Pu et al. [27] investigated the correlation between lateral tibial plateau CT imaging parameters and lateral meniscus injury through arthroscopy in 296 patients with Schatzker II fractures. They found a higher possibility of lateral meniscus injury when LPD exceeds 9 mm and/or LPW more than 7.5 mm. In summary, our findings generally support previous studies, indicating that both LPD and LPW are important indicators for preoperative prediction of associated soft tissue injuries. However, our study suggests minimum LPD and LPW values of 8.40 mm and 7.90 mm, respectively, for predicting lateral meniscus injury. Our research specifically emphasizes the strong association between lateral plateau displacement and lateral meniscus injury in Schatzker IV-C fractures for the first time. Inconsistencies with previous studies’ result may stem from variations in factors such as Included research subjects, patient Schatzker classification, measurement methods, and sample sizes.

There are several limitations in the current study. Firstly, manual measurements have inherent subjectivity, which may introduce potential bias into the results. Secondly, we included a relatively small number of patients with Schatzker IV-C fractures which may introduce bias into our results. Thirdly, we were unable to differentiate degenerative or traumatic meniscal lesions. Thus, whether existing pre-existing meniscus injury prior to the fracture may cannot completely guarantee. Lastly, this study did not address the specific location and classification of meniscal injuries.

## Conclusions

Schatzker IV-C tibial plateau fracture was accompanied by a high risk of lateral meniscal injury when LPD exceeds 8.40 mm and/or LPW exceeds 7.90 mm. Meanwhile, our results may better explain the correlation between Schatzker IV-C tibial plateau fracture and soft tissue injury and provide potential predictors (LPD and LPW) for early diagnosing Schatzker IV-C tibial plateau fracture-related meniscus injury. Additionally, our findings may enable orthopedic surgeons to anticipate lateral meniscal injuries in Schatzker IV-C tibial plateau fractures through the numerical value of LPD and LPW measured by preoperative coronal CT scans.

### Electronic supplementary material

Below is the link to the electronic supplementary material.

Supplementary Material 1

Supplementary Material 2

## Data Availability

The datasets generated and analyzed during the current study are not publicly available due to ethical restrictions regarding patient data and anonymity, but may be available from the corresponding author upon reasonable request.

## Abbreviations

LPD: Lateral plateau depression
LPW: Lateral plateau widening
ORIF: Open reduction internal fixation
ROC: Receiver operating characteristic
AUC: Area under the curve
