# Supplementary material for: Correlation of preoperative CT imaging shift parameters of the lateral plateau with lateral meniscal injury in Schatzker IV-C tibial plateau fractures
Source: BMC Musculoskelet Disord. 2023 Oct 6;24:793. doi: 10.1186/s12891-023-06924-7 (PMC10557321; doi:10.1186/s12891-023-06924-7)
Supplement: Supplementary file 1 — Supplementary Material 1 [file 12891_2023_6924_MOESM1_ESM.docx]

Table S1. The detailed information regarding local soft tissue injuries of Schatzker IV-C tibial plateau fractures patients.

| **Injury types and treatment methods** | **Patients (N=60)** |
| --- | --- |
| **Concomitant anterior cruciate ligament (ACL) injury** | 52(86.7%) |
| 1. Hollow screws fixation for ACL avulsion fractures | 4(7.7%) |
| 1. Conservative treatment | 48(92.3%) |
| **Concomitant posterior cruciate ligament (PCL) injury** | 38(63.3%) |
| 1. Conservative treatment | 38(100%) |
| **Concomitant medial meniscus injury** | 25(41.7%) |
| 1. Conservative treatment | 25(100%) |
| **Concomitant lateral collateral ligament (LCL) injury** | 39(65%) |
| 1. Anchor fixation for bony avulsion injuries | 8(20.5%) |
| 1. Conservative treatment | 31(79.5%) |
| **Concomitant medial collateral ligament (MCL) injury** | 19(31.7%) |
| 1. Conservative treatment | 19(100%) |
