# Supplementary material for: Correlation of preoperative CT imaging shift parameters of the lateral plateau with lateral meniscal injury in Schatzker IV-C tibial plateau fractures
Source: BMC Musculoskelet Disord. 2023 Oct 6;24:793. doi: 10.1186/s12891-023-06924-7 (PMC10557321; doi:10.1186/s12891-023-06924-7)
Supplement: Supplementary file 2 — Supplementary Material 2 [file 12891_2023_6924_MOESM2_ESM.docx]

Table S2. The results of 60 patients with Schatzker IV-C tibial plateau fractures one-year postoperative Hospital for Special Surgery (HSS) Knee Score

| **HSS Score** | **Patients (N=60)** |
| --- | --- |
| Excellent (>85 points) | 8(13.33%) |
| Good (70-84 points) | 29(48.33%) |
| Moderate (60-69 points) | 18(30.0%) |
| Poor (<59 points) | 5(8.33%) |
